# Genome Sequence of OXA-726-Encoding Aeromonas dhakensis Igbk (Sequence Type 1171) from an Edible Snail Traded in Nigeria

**DOI:** 10.1128/mra.00343-22

**Published:** 2022-05-31

**Authors:** Arthur C. Okafor, Frank C. Ogbo, Adriana Cabal Rosel, Anna Stöger, Fred C. Akharaiyi, Beatriz Prieto, Franz Allerberger, Werner Ruppitsch

**Affiliations:** a Institute of Medical Microbiology and Hygiene, Austrian Agency for Health and Food Safety, Vienna, Austria; b Department of Applied Microbiology and Brewing, Nnamdi Azikiwe University, Awka, Nigeria; c Department of Microbiology, Edo State University, Uzairue, Iyamho, Nigeria; University of Maryland School of Medicine

## Abstract

Aeromonas dhakensis is the most virulent *Aeromonas* species pathogenic to both animals and humans. The degree of its risk to health seems masked by misidentifications. We present the genome sequence of A. dhakensis Igbk (sequence type 1171), associated with snails, harboring the OXA-726 gene in the chromosome.

## ANNOUNCEMENT

Aeromonas dhakensis is an important pathogen isolated from the environment (1, 2), animals (3, 4), and humans (5, 6). It was first isolated from children with diarrhea (5). A. dhakensis is frequently misidentified as Aeromonas hydrophila by phenotypic methods and matrix-assisted laser desorption ionization–time of flight mass spectrometry (MALDI-TOF MS) (7, 8). A strain of A. hydrophila (GenBank accession number MK530176) that was recently identified from edible snails in a different study in Nigeria using 16S rRNA gene sequencing (9) might also have been misidentified due to the strong similarities of the 16S rRNA gene.

Here, we report the genome sequence of an OXA-726-encoding A. dhakensis isolate that was obtained in October 2021 from a raw snail (Achatina achatina) traded in the wet market at Igboukwu Town, southeast Nigeria. An intestinal section (50 g) was aseptically collected and homogenized with 450 mL of peptone water (Oxoid, Hampshire, UK); aliquots were enriched for 24 h in nutrient broth (Oxoid) containing 3% NaCl and were subsequently plated on thiosulfate-citrate-bile salts-sucrose agar (Titan, Delhi, India) with incubation at 35°C for 24 h. Presumptive colonies were subcultured on blood agar (bioMérieux, Marcy-I’Etoile, France) for 24 h at 35°C. MALDI-TOF MS (Bruker, Bremen, Germany) with Biotyper software v4.1.100 was used for identification of the isolate. Genomic DNA was extracted using the MagAttract high-molecular-weight (HMW) DNA kit (Qiagen, Hilden, Germany). Genomic libraries were prepared using the Nextera XT kit (Illumina, San Diego, CA, USA) and sequenced (2 × 300-bp reads) on a MiSeq instrument. Raw reads were assembled using SPAdes v3.15.2 (https://cab.spbu.ru/software/spades) and quality controlled using FastQC v0.11.7 and Trimmomatic v0.36. Whole-genome-based taxonomic analyses were performed using digital DNA-DNA hybridization (dDDH) (https://tygs.dsmz.de) and the Tetra Correlation Search (TCS) (http://jspecies.ribohost.com/jspeciesws). MALDI-TOF MS identified our strain as A. hydrophila, whereas dDDH and TCS identified it as A. dhakensis (93.9% and 0.99988 Z-score identity, respectively, with respect to A. dhakensis CIP 107500). A multilocus sequence typing (MLST) scheme was engaged to assign a new sequence type (ST), ST-1171, to A. dhakensis strain Igbk. An *ad hoc* core genome MLST scheme with 2,928 targets was created with Seqsphere+ v8.3.1 (Ridom, Münster, Germany) and used for comparison with all A. dhakensis strains available from GenBank. This comparison showed 669 allelic differences from a A. dhakensis strain isolated in 2019 from a rectal swab sample in Thailand. The NCBI Prokaryotic Genome Annotation Pipeline (PGAP) was used for annotation (10). Genome data for A. dhakensis Igbk are presented in Table 1. The strain was further characterized using the Comprehensive Antibiotic Resistance Database (CARD), MobileElementFinder (https://cge.cbs.dtu.dk/services/MobileElementFinder), and PathogenFinder (https://cge.cbs.dtu.dk/services/PathogenFinder). Default parameters were used for all software except where otherwise noted. Our strain harbored a range of antimicrobial genes (Table 1), notably *bla*_OXA-726_, which codes for a type of class D β-lactamase purported to be a looming global public health threat (11). Virulence genes and plasmids were not detected. The pathogenic probability was 0.771, matching 31 pathogenic gene families. The presented features of A. dhakensis Igbk (ST-1171) will contribute data useful to public health authorities for comparative genomics.

**TABLE 1:** Genomic features of Aeromonas dhakensis Igbk (ST-1171)

Attribute	Finding
Assembly size (bp)	4,829,827
No. of reads	1,255,396
Avg read length (bp)	252
No. of contigs	260
*N*_50_ (bp)	80,959
GC content (%)	61.6
Genome coverage (×)	57
Total no. of genes	4,585
No. of coding genes	4,380
No. of RNA genes	155
No. of noncoding RNA genes	7
No. of pseudogenes	50
Antimicrobial resistance genes	*bla*_OXA-726_, *adeF*, *rsmA*, *imiH*, EF-Tu, *ampH*, *cphA8*

### Data availability.

This whole-genome shotgun project has been deposited in DDBJ/ENA/GenBank under the accession number JALGBK000000000 (BioProject accession number PRJNA819262 and BioSample accession number SAMN26920427). This is the first version of this genome. The raw sequence reads have been deposited in the Sequence Read Archive (SRA) under the accession number SRR18516351.

## References

[1] Martinez-Murcia AJ, Saavedra MJ, Mota VR, Maier T, Stackebrandt E, Cousin S. 2008. *Aeromonas aquariorum* sp. nov., isolated from aquaria of ornamental fish. Int J Syst Evol Microbiol 58:1169–1175. doi:10.1099/ijs.0.65352-0. 18450708

[2] Esteve C, Alcaide E, Blasco MD. 2012. *Aeromonas hydrophila* subsp. *dhakensis* isolated from feces, water and fish in Mediterranean Spain. Microbes Environ 27:367–373. doi:10.1264/jsme2.me12009. 22472298 PMC4103543

[3] Yano Y, Hamano K, Tsutsui I, Aue-Umneoy D, Ban M, Satomi M. 2015. Occurrence, molecular characterization, and antimicrobial susceptibility of *Aeromonas* spp. in marine species of shrimps cultured at inland low salinity ponds. Food Microbiol 47:21–27. doi:10.1016/j.fm.2014.11.003. 25583334

[4] Pu W, Guo G, Yang N, Li Q, Yin F, Wang P, Zheng J, Zeng J. 2019. Three species of *Aeromonas* (*A. dhakensis*, *A. hydrophila* and *A. jandaei*) isolated from freshwater crocodiles (*Crocodylus siamensis*) with pneumonia and septicemia. Lett Appl Microbiol 68:212–218. doi:10.1111/lam.13112. 30609084

[5] Huys G, Kampfer P, Albert MJ, Kuhn I, Denys R, Swings J. 2002. *Aeromonas hydrophila* subsp. *dhakensis* subsp. nov., isolated from children with diarrhoea in Bangladesh, and extended description of *Aeromonas hydrophila* subsp. *hydrophila* (Chester 1901) Stanier 1943 (approved lists 1980). Int J Syst Evol Microbiol 52:705–712. doi:10.1099/00207713-52-3-705. 12054229

[6] Huang M, Chen H, Li C, Liu Y, Gan C, El-Sayed Ahmed MAE-G, Liu R, Shen C, Zhong R, Tian G-B, Huang X, Xia J. 2020. Rapid fulminant progression and mortality secondary to *Aeromonas dhakensis* septicemia with hepatitis B virus infection following the ingestion of snakehead fish in mainland China: a case report. Foodborne Pathog Dis 17:743–749. doi:10.1089/fpd.2019.2780. 32985901

[7] Figueras MJ, Alperi A, Saavedra MJ, Ko WC, Gonzalo N, Navarro M, Martínez-Murcia AJ. 2009. Clinical relevance of the recently described species *Aeromonas aquariorum*. J Clin Microbiol 47:3742–3746. doi:10.1128/JCM.02216-08. 19741075 PMC2772615

[8] Kitagawa H, Ohge H, Yu L, Kayama S, Hara T, Kashiyama S, Kajihara T, Hisatsune J, Sueda T, Sugai M. 2020. *Aeromonas dhakensis* is not a rare cause of *Aeromonas* bacteremia in Hiroshima, Japan. J Infect Chemother 26:316–320. doi:10.1016/j.jiac.2019.08.020. 31570322

[9] Okafor AC. 2019. Studies on bacterial hazards associated with edible land snails (*Achatina achatina*) from south east Nigeria. PhD thesis. Nnamdi Azikiwe University, Awka, Nigeria. https://phd-dissertations.unizik.edu.ng/onepaper.php?p=504.

[10] Tatusova T, DiCuccio M, Badretdin A, Chetvernin V, Nawrocki EP, Zaslavsky L, Lomsadze A, Pruitt KD, Borodovsky M, Ostell J. 2016. NCBI Prokaryotic Genome Annotation Pipeline. Nucleic Acids Res 44:6614–6624. doi:10.1093/nar/gkw569. 27342282 PMC5001611

[11] Pitout JD, Peirano G, Kock MM, Strydom KA, Matsumura Y. 2019. The global ascendency of OXA-48-type carbapenemases. Clin Microbiol Rev 33:e00102-19. doi:10.1128/CMR.00102-19. 31722889 PMC6860007

